# Thyroid Hormones Regulate Zebrafish Melanogenesis in a Gender-Specific Manner

**DOI:** 10.1371/journal.pone.0166152

**Published:** 2016-11-10

**Authors:** Raúl Guillot, Borja Muriach, Ana Rocha, Josep Rotllant, Robert N. Kelsh, José Miguel Cerdá-Reverter

**Affiliations:** 1 Department of Fish Physiology and Biotechnology, Instituto de Acuicultura de Torre de la Sal, Consejo Superior de Investigaciones Científicas, (IATS-CSIC), Ribera de Cabanes, Castellón, Spain, 12595; 2 Facultad Ciencias de la Salud, Universidad CEU Cardenal Herrera, Castellón, Spain, 12006; 3 Aquatic Molecular Pathobiology Group, Instituto de Investigaciones Marinas, Consejo Superior de Investigaciones Científicas, (IIM-CSIC), Vigo, Spain, 36208; 4 Centre for Regenerative Medicine and Developmental Biology Programme, Department of Biology and Biochemistry, University of Bath, Bath, England BA2 7AY; Chinese Academy of Sciences, CHINA

## Abstract

Zebrafish embryos are treated with anti-thyroidal compounds, such as phenylthiourea, to inhibit melanogenesis. However, the mechanism whereby the thyroidal system controls melanin synthesis has not been assessed in detail. In this work, we tested the effect of the administration of diets supplemented with T3 (500μg/g food) on the pigment pattern of adult zebrafish. Oral T3 induced a pronounced skin paling in both adult female and male zebrafish that was reversible upon cessation of treatment. The number of visible melanophores was significantly reduced in treated fish. Accordingly, treatment down-regulated expression of tyrosinase-related protein 1 in both sexes. We also found sexually dimorphic regulation of some melanogenic genes, such as *Dct*/*Tyrp2* that was dramatically up-regulated in females after T3 treatment. Thus, we demonstrated that melanogenesis is reversibly inhibited by thyroid hormones in adult zebrafish and make the discovery of gender-specific differences in the response of melanogenic gene expression. Thus, fish gender is now shown to be an important variable that should be controlled in future studies of fish melanogenesis.

## Introduction

Fish exhibits a wide chromatic diversity that is obtained by the patterned distribution of different types of chromatophores that can be divided mainly into light-absorbing (melanophores, xantophores, erythrophores and cyanophores) and light-reflecting (leucophores and iridophores) chromatophores [1]. The pigment pattern of zebrafish is obtained by the patterned distribution of three different chromatophore types, i.e. melanophore, xanthophore and iridophore. In the dark stripes xanthophores occupy the most superficial hypodermal layer which is underlaid by type S iridophores. Just beneath these, a layer of melanophores is found on a deepest layer of type L iridophore, immediately above the skeletal muscle. In the interstripe region, type S iridophores lay just above the muscular layer while xhantophores are found between the tractum compactum of the dermis and the iridophore layer [2]. Pigment pattern in adult zebrafish is sexually dimorphic. Adult males exhibit a yellow shade that is less intense in females, while females are brighter than males. No sex differences in the patterned distribution of chromatophore have been reported [2,3] Therefore, gender differences in zebrafish pigmentation might be expected to result from differences in the ratio of chromatophore types and/or the quantity of pigments. It is thus conceivable that genes involved in pigment synthesis exhibit gender-specific regulation but this assumption is obviated in many experimental designs.

The synthesis of melanin is limited by the hydroxylation of tyrosine to dopaquinone mediated by tyrosinase (Tyr) activity. Dopaquinone is converted into dopachrome that serves as a substrate for tyrosinase-related protein 2 (Tyrp2) to catalyze the formation of 5,6 dihydroxyindole-2-carboxilic acid (DHICA). Tyrosinase-related protein 1 (Tyrp1) mediates the last step of melanogenesis by oxidizing DHICA to melanin [4]. Anti-thyroidal compounds, such as phenylthiourea (PTU), are used commonly to prevent melanisation during embryogenesis by blocking all tyrosinase-dependent steps in the melanin pathway [5]. Recent investigations have related the thyroidal system to the regulation of melanin synthesis in fish [6,7]. *Tyr* gene expression is down-regulated in zebrafish embryos showing low intracellular 3,3',5-triiodo-L-thyronine (T3) availability but exogenous T3 causes increased pigmentation thus suggesting that the activation of the thyroidal system is necessary for the regulation of the melanisation in early larvae [6,7]. On the contrary, T3 exposition or endocrine disruptors that mimic thyroid hormone activity decreased melanin pigmentation and increased apoptosis in the retina of zebrafish embryos [8]. Accordingly, McMennamin and collaborators [9] have shown that treatment with thyroxine (T4), as well as an activating mutation in the *thyroid-stimulating hormone receptor* (*tshr/opallus*) gene, results in a paling phenomenon. Recently, we have demonstrated the presence of thyrogenic activity in commercially available fish diets that are widely used in aquaculture (Quesada et al., 2012). It is therefore plausible that the presence of thyrogenic activity in fish feeds could produce pigmentation anomalies in reared fish. In fact, dietary-induced pigment anomalies are common in reared flatfish including albinism or pseudoalbinism of the ocular side and hypermelanism of the blind side [10] (Darias et al., 2013).

In a different study focusing the thyroidal regulation of melanocortin accessory proteins (MRAPs), we observed that adult zebrafish treated with oral T3 undergo a profound skin paling, [11] opposite to that reported in embryos [6,7]. Therefore, we design a new experiment to quantify the effect of thyroid hormones on different genes involved in the control of melanogenic pathway but also involved in the melanophore differentiation. Here, we demonstrated that melanogenesis is reversibly inhibited by thyroid hormones in adult zebrafish. We found the existence of both gender-specific patterns and response to thyroidal treatments of the melanogenic gene expression. Thus, our results demonstrate that fish gender is a critical variable that should be controlled in studies of fish melanogenesis.

## Material and Methods

### Animals and reagents

Wild-type TU strain one year old zebrafish were raised at 24–28°C, with 14h light/10h dark cycle. All experiments were carried out in accordance with the principles published in the European animal directive (86/609/EEC) and approved by Consejo Superior de Investigaciones Cientifícas (CSIC) ethics committee (Project Number AGL2013-46448-C3-3-R) as well as the local ethics committee at the Instituto de Acuicultura de Torre de la Sal. Unless otherwise indicated, all reagents were purchased from Sigma (St Louis MO, USA).

### Diets and feeding protocol

Animals were fed with control diet (CTRL), a granulated commercial diet [Supervit (Tropical, DE)]. To prepare the experimental diet, the amount of T3 to reach the experimental dose (500μg/g food) was dissolved in 2 ml 100% ethanol and sprayed onto 10 g of control diet, mixed, dried at room temperature and stored at 4° C. Animals were fed 4% of body weight (BW) with control or T3-containing diet. The total amount of food was divided into two meals provided at 10.00h and 14.00h. Tank water was renovated every three days.

### Hormonal levels

After euthanization by overdose of anesthetic (MS-222) at 10 am, zebrafish blood from 15 CTRL (7 females and 7 males) and T3-treated (7 females and 7 males) zebrafish was collected by sectioning the caudal fin at peduncular level. Serum T3 levels were measured by ELISA (Abcam, UK) according to the manufacturer’s instructions.

### Sex effects on pigmentation-related genes

The whole skin of twenty adult zebrafish (10 males and 10 females) was sampled for total RNA extraction and gene expression experiments. After euthanization at 10 am by overdose of anesthetic (MS-222) and skin sampling, gonads were dissected to confirm the fish gender. Skin samples were kept at -80°C until processing for RNA extraction.

### Effects of T3 on pigmentation-related genes

Ninety adult zebrafish (BW = 0.33 ± 0.05 g) were reared in four 40-liter tanks. Two tanks (n = 20 each) were fed with CTRL diet whereas the other two tanks (n = 25 each) were fed with T3-containing diet. After 7 days, animals were euthanized at 10 am as before and the whole skin was removed from 37 (19 CTRL and 18 T3-treated fish) animals and processed for total RNA extraction (see below). The same protocol was followed for 8 further days (15 day treatment in total) and skin samples were dissected from 27 animals (13 CTRL and 14 T3-treated fish) with the same aim following the previous protocol. Gonads were extracted for sexing. To study the reversibility of the T3 effect, 18 fish that had been hormone-treated for 15 days were subsequently fed with CTRL diet for a further 15 days. The experiment was done independently three times to corroborate the morphological effects, but tissue samples were only obtained in the first experiment.

### Melanophore counts

**Fig 1:**
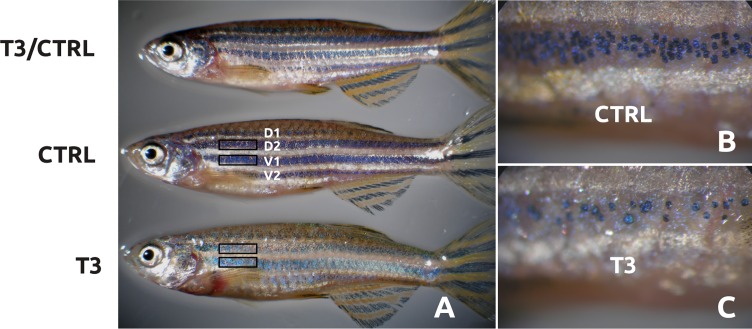
Panel A shows the effects of oral T3 (500 μg/g of food) on zebrafish pigment phenotype. Fish were feed with control (CTRL) or T3-supplemented (T3) diet during 15 days and then fed with CTRL diet for 15 additional days (T3/CTRL). Higher magnification of melanic stripes in CTRL (B) and T3 (C) fish. D1 and D2, dorsal stripes 1 and 2. V1 and V2, ventral stripes 1 and 2, respectively. Boxes represent melanophore counting areas in D2 and V1.

To enable accurate quantification of melanophore numbers, twenty fish (10 males and 10 females from each treatment) were treated with epinephrine (10 mg/ml) for approximately 30 min. Afterwards, fish were sacrificed by anesthetic overdose, fixed in 4% paraformaldehyde (PFA) in phosphate buffer 0.1M, pH = 7.4 and imaged using an Olympus SZX16 stereo microscope. Fixation with PFA removes the pigments of xanthophores and iridophores but does not affect melanin. Melanophores within a 1 mm^2^ area on the rostral portion of the second (D2) dorsal stripe as well as the first (V1) and second (V2) ventral stripe (Fig 1) were counted manually, from a lateral view, utilizing Adobe Photoshop CS2 software. The number of melanophores was plotted against the area in mm^2^.

### RNA isolation, RT-PCR and qRT-PCR

Total skin RNA was purified with Tri-Reagent and 1μg was used for cDNA synthesis with Superscript III reverse transcriptase (Invitrogen) primed with random hexamers and oligo(dT)12-18 (Invitrogen). The cDNA was subsequently used as template for quantitative real-time PCR (qPCR). The expression of genes encoding melanogenic enzymes [Tyrosinase (*Tyr*), Tyrosinase-related protein 1a (*Tyrp1a*), Tyrosinase-related protein 1b (*Tyrp1b*), Dopachrome tauromerase (*Dct or Tyrp2*), transcription factors [Microphthalmia-associated transcription factor a (*Mitfa* or *nacre*), Sox10 (*sox10 or colourless*), Forkhead transcription factor 3 (*Foxd3*)], receptors [Kit receptor tyrosine kinase a (*Kita*, *sparse*), Kitb (*kitb*), Melanocortin 1 receptor (*Mc1r*)], ligands [(Agouti-signaling protein 1 (*Asip1*)] and carriers [Solute carrier family 24 member 5 (*Slc24a5*)] was evaluated by qRT-PCR. Primer and cDNA concentrations were tested for each gene and conditions with reaction efficiencies below 90% were not accepted. One microlitre of pure or diluted cDNA was added to 10 μl of 2X Taqman PCR master mix (ABgene, Thermo Scientific, Spain). Reactions were carried out in triplicate in a Realplex Mastercycler (Eppendorf, Spain). The housekeeping genes *β-actin* and *elongation factor-1alpha (EF-1α)* were used as internal reference to normalize the cDNA template between samples. Normalized relative quantities of mRNA expression were calculated with the mathematical method of ΔΔCt. The melting curves of the products were verified to confirm the specificity of PCR products. Primer sequences are shown in S1 Table.

### Data analysis and statistics

Statistical analysis was conducted by t-test, one -way ANOVA followed by Tukey-HSD's multiple range test and two-way ANOVA. Differences considered significant when p<0.05 for serum hormone levels, p<0.004 for gene expression and p<0.01 for melanocyte counts after Bonferroni's correction of P value for multiple tests performed simultaneously on a single data set. Data were tested previously for normality (Kolmogorov–Smirnov's test) and variance homogeneity (Bartlett's test).

## Results

### Sex-dependent expression of genes involved in melanogenesis

**Fig 2:**
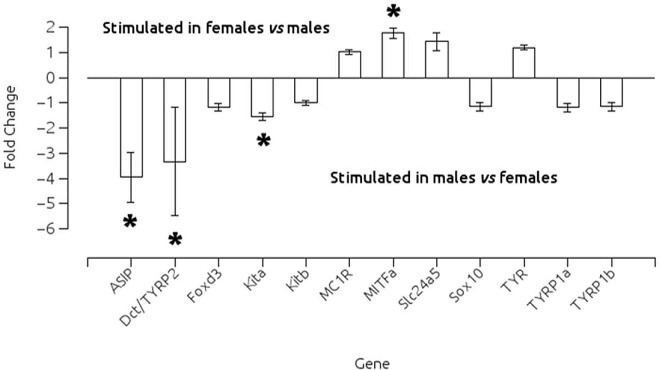
Effects of gender on gene expression levels of some genes associated with the melanogenic pathways in zebrafish as measured by qPCR. *Tyrosinase* (*tyr*), *tyrosinase-related protein 1a* (*tyrp1a*), *tyrosinase-related protein 1b* (*tyrp1b*), *dopachrome tautomerase* (*dct*/*tyrp2*), *microphthalmia-associated transcription factor a* (*mitfa*/*nacre*), *sox10* (*colourless*), *forkhead transcription factor 3* (*foxd3*), *kit receptor tyrosine kinase a* (*kita*, *sparse*), *kitb*, *melanocortin 1 receptor* (*mc1r*), *agouti-signaling protein 1* (*asip1*) and *solute carrier family 24 member 5* (*slc24a5*). Asterisks indicate significant differences after t-test (p<0.01) between sexes after Bonferroni's correction.

Prior to studying the gender-specific effects of T3 treatment on adult zebrafish, we set up a preliminary experiment to evaluate the differences in the expression of genes involved in melanophore development and melanin synthesis inherent to gender *per se*. The expression of *asip1*, *dct*/*tyrp2* and *kita*/*sparse* was significantly higher in males compared with females, whereas expression of *mitfa*/*nacre* was significantly higher in females when compared to males (Fig 2).

### T3 plasma levels

**Fig 3:**
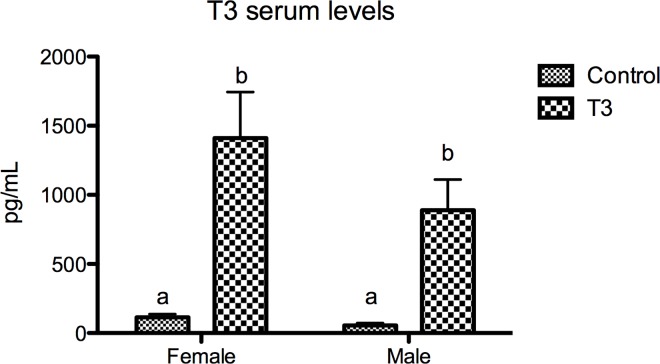
Serum T3 levels after hormone oral administration for 15 days. Different letters indicate significant differences after one way-ANOVA followed by Tukey’s multiple range test (p<0.05). No significant interactions gender/treatement were detected after two way-ANOVA (p<0.05).

Oral hormone administration resulted into increased T3 serum levels in both males and females whne compared to control animals. No differences in serum levels were detected between treated males and females (Fig 3).

### T3-induced pigment pattern is gender-specific

**Fig 4:**
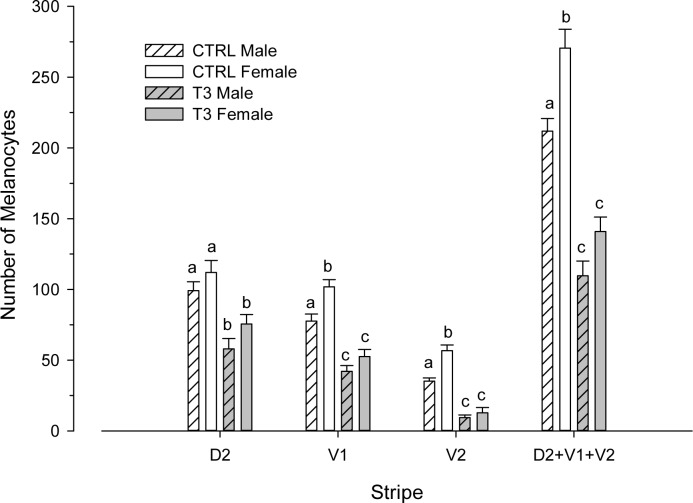
Number of visible melanised melanocytes after oral supplementation of control (CTRL) diet with T3. Melanophore counts within a 1 mm^2^ area on the rostral portion of the second dorsal (D2) and first ventral (V1) stripes were counted manually, from a lateral view. Different letters indicate significant differences in the number of melanophores for each stripe after one-way ANOVA followed by Tukey-HSD test (p<0.05).

Previous studies demonstrated that oral T3 induced skin paling in zebrafish. Here, we wished to refine and extend these studies by assessing them for gender specific effects on melanogenic gene expression. One-way ANOVA showed that females always have more melanocytes in the ventral stripes than males and the same is true when stripes were considered altogether (Fig 4). Oral administration of T3 severely inhibited melanogenesis in zebrafish males and females. Skin paling was observed visually at 7 days (data not shown) and notably evident after 15 days of treatment (Fig 1). When T3 treatment ceased the phenotype recovered to a normal pigmentation level within 15 days (Fig 1). Two-way ANOVA revealed both sex and treatment effects on the number of visible melanophores/mm^2^ in V1, V2 and D2+V1+V2 (S2 Table) but interactions sex/treatment only reached significant values in V2. However, sex did not induce differences in D2 (p = 0.039) as T3 treatment did (p<0.001).

### Gender-specific effects of T3 on genes involved in melanogenesis

**Fig 5:**
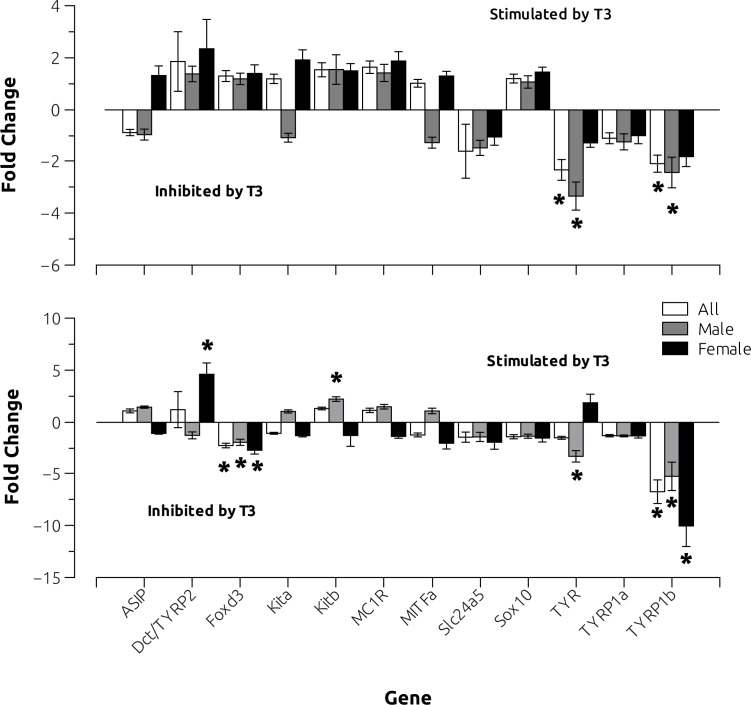
Effects of oral T3 (500 μg/g of food, B) on gene expression levels of genes associated with the melanogenic pathways in zebrafish as measured by qPCR after 7 days (upper panel) and 15 days (lower panel). After 7 days post-treatment, we found 10 and 9 control females and males, respectively and 10 and 8 T3-treated females and males, respectively. After 15 days post-treatment, we found 6 and 7 control males and females, respectively and 8 and 6 T3-treated males and females, respectively. Asterisks indicate significant T3-induced differences after t-test (p<0.004) after Bonferroni's correction.

To look for subtler effects and to begin to explore possible mechanisms of paling, we turned to qRT-PCR and asked whether T3 treatment affected melanophore gene expression in a gender-specific way. First, we compared the expression of the pigmentation-related genes between both sexes of control animals and found similar results to those reported above (data not shown). We then assessed whether T3 treatment differentially affected melanophore gene expression in a gender-specific way at 7 and 14 days (Fig 5). We found both sex-dependent and sex-independent effects of T3-treatment on the expression of pigmentation-related genes. The pattern of sex-dependent effects was complex. Thus, after 7 days, T3 treatment *tyr* and *tyrp1b* expression was down-regulated in treated males (but not females) (Fig 5). After 15 days, *dct/tyrp2* was, respectively, -upregulated in females, but not significantly changed in males; in contrast, *kitb* and *tyr* were, respectively, up- and down-regulated in males, but unchanged in females (Fig 5). Two-way ANOVA revealed significant interactions treatment/gender in the expression levels of *Dct*, and *Tyr* genes after 15 days treatment (S3 Table). Gender-independent effects were also seen. Thus, at 15 days, T3-treatment inhibited *tyrp1b* and *foxd3* expression in the skin of both males and females (Fig 5).

## Discussion

In this study, our most striking conclusion is that there are previously overlooked gender-specific differences in pigmentation, and in melanogenic gene expression patterns, in adult zebrafish. Although many zebrafish workers use body pigmentation as a guide to sexing, the emphasis is usually on the more intense yellow (xanthophore) colouration of males; the somewhat less contrasting pattern of the stripes in females is known, but whether this reflects differences in melanophore biology, or is somehow an optical illusion resulting from the xanthophore colouration has not been widely considered. Our data clearly show differences in melanophore biology in male and female zebrafish. This has important implications for studies of adult zebrafish pigmentation, since gender now becomes an important factor to control during such studies.

We demonstrate that oral administration of T3 induces skin paling by reducing the number of melanised melanophores in the zebrafish stripes. Recently, McMennamin [9] have shown that treatment with thyroid hormones result in a paling phenomenon as described here; in agreement with our study, they also show that the paling results from a decreased number of melanophores, correlating with increased melanophore death in hyperthyroid animals. A full explanation will require a more comprehensive characterization of gene expression in the skin, but our data suggest the hypothesis that inhibition of Tyrp1b expression in the skin may well contribute to this paling, since this is one effect that is consistent in fish of both genders. A dominant Tyrp1a mutation leads to melanophore death in zebrafish, perhaps through disrupting melanosome integrity [12], but unfortunately the zebrafish *tyrp1b* mutant phenotype remains to be characterized. It will be important to test whether simple loss of function mutations result in decreased intensity of melanin pigment, similar to the effect seen in mouse [13]. The expression of *foxd3* was also consistently downregulated in both males and females. Foxd3 is a robust marker of pre-migratory neural crest throughout vertebrates [14]. This transcription factor acts as a transcriptional repressor in the neural crest that is associated with the maintenance of pluripotency and pluripotent cells. In zebrafish embryos, it prevents melanophore fate by inhibiting *mitfa* expression [14,15]. *Foxd3* is also expressed in putative glial cells of the peripheral nervous system [16]. Pigment cells in adult fish derive from post-embryonic stem cells of neural crest origin [17,18], associated with the peripheral nerves and glia [19, 20]. During post-embryonic development, a proliferative population of Erbb3b-dependent *foxd3*- and *sox10*-expressing cells associated with the peripheral nervous system differentiates into adult melanophores [19, 20]. It is conceivable that the T3-induced *foxd3* inhibition may reflect changes in the balance of melanocyte stem cell proliferation/differentiation, but detailed investigation will be necessary to explore this idea. *Foxd3* inhibition could result from a feedback mechanism uncovered by the hormonally-induced inhibition of the melanogenesis pathway.

The effects of T3 treatment appear to vary with age. T3 treatment promotes melanisation in zebrafish embryos [6], whereas morpholino-mediated knockdown of type 2 iodothyronine deiodinase (D2), delays embryonic pigmentation [7]. *Tyr* expression is inhibited in D2 morphants suggesting that T3 is required for the normal expression of the melanogenic enzyme in early developmental stages. T3 treatment of D2 morphants rescues the pigment phenotype as well as normalizing *tyr* expression [7]. These results are opposite to those in our experiments however careful observation of the photographic data reported after T3 treatment in zebrafish larvae [6] suggests that T3 supplementation seems to stimulate pigmentation at 36 hours post fertilization (hpf). This phenotype is no longer visible at 72 hpf. Actually, it seems that the number of melanophores is reduced at 72hpf. Unfortunately, no quantitative studies were conducted in these studies [6]. In addition, an earlier study reported that the paling phenomenon can be reversibly induced by thyroid hormones during metamorphosis, [21]. It is thus likely that the ability of thyroid hormones to induce paling appears sometime during metamorphosis.

The paling effect is more severe in females than in males as demonstrated by significant positive sex/treatment in V2 suggesting gender-specific contributions to the molecular mechanism. We observe a complex series of gender-independent and gender-specific regulatory changes in melanogenic gene expression but these changes cannot be attributed to gender differences in T3 metabolism since both treated male and females displayed similar serum levels after hormonal treatment. The differential expression of *tyr*, and *dct/tyrp2* demonstrate a sexually-dimorphic response of the melanogenic pathway to T3 treatment. Again, these data highlight the importance of sex in the study of adult melanogenesis in zebrafish. Can anything be said regarding the molecular mechanisms of paling and the sexual dimorphism in the severity? Our data suggests that this may come from gender specific effects on gene expression. Consistent with the T3-induced phenotype, *tyr* expression was downregulated but only in males. Since Tyrp1 activity is downstream of Tyr activity a more severe phenotype is not expected in males. However, downregulation of *tyrp1b* expression was more severe in T3-treated females, despite it did not reach statistical significance after Bonferroni’s correction (p = 0.03), matching the increased T3 effects in reducing of number of visible melanophores. Unexpectedly, *dct/tyrp2* expression was stimulated only in females. We do not have an explanation for the gender-specific stimulation of *dct/tyrp2* expression and more experiments are required to understand the role of the T3-stimulated *dct/tyrp2* expression levels.

In summary, we demonstrate that administration of thyroid hormones inhibits zebrafish melanogenesis due to a significant decrease of melanised melanophores and a robust decrease of *tyrp1* expression in both males and females. This effect is reversible, since cessation of the T3 treatment reverses the pigmentation anomalies back to the control phenotype, suggesting that maintenance of the adult pigment pattern is hormonally controlled. We demonstrated that some key genes related to melanogenesis are differentially expressed in males and females thus highlighting the importance of gender in the regulation of melanogenic pathways in zebrafish. Our demonstration that the response to T3 treatment is sexually dimorphic will only be explained by detailed characterization of the adult melanophore gene regulatory network. Meanwhile, our data highlight the importance of taking account of gender effects when studying adult zebrafish melanogenesis.

## Supporting Information

S1 Table Primers used for qPCR. Primer sequences used for quantification of pigmentation-related genes in zebrafish. Asip1 (agouti-signaling protein 1), Dct (dopachorme tautomerase or tyrosinase-related protein 2), FoxD3 (forkhead box D3), cKit (kit receptor tyrosine kinase type a or CD117), Mc1r (melanocortin receptor type 1), Mitfa (microphthalmia-associated transcription factor type a), Slc24a5 (solute carrier transporter 24a member 5), Sox10 (transcription factor Sox-10), Tyr (tyrosinase), Tyrp1a (tyrosinase-related protein 1 type a), Tyrp1b (tyrosinase-related protein 1 type b). See material and method for details.
(DOCX)

S2 Table Comparisons on the number of melanophores. Two-Way ANOVA table for comparisons on the number of melanophores in the Dorsal 2 (D2), Ventral 1 (V1), Ventral 2 (V2) or Dorsal 2 + Ventral 1+Ventral 2 (D2+V1+V2) stripes after T3 treatment according to fish gender. Differences (bold numbers) were considered significant when p<0.01 after Bonferroni's correction for multiple tests. See material and method for details.
(DOCX)

S3 Table Comparisons on the expression of melanophore-related genes. Two-Way ANOVA table for comparisons on the expression of melanophore-related genes after 7 and 15 days of T3 treatment according to fish gender. Differences (bold numbers) were considered significant when p<0.004 after Bonferroni's correction for multiple tests. See material and method for details.
(DOCX)

S1 Text Data on number of Melanopohore. number of melanophores in the Dorsal 2 (D2), Ventral 1 (V1), Ventral 2 (V2) or Dorsal 2 + Ventral 1+Ventral 2 (D2+V1+V2) stripes after T3 treatment according to fish gender. See material and method for details.
(XLSX)

S2 Text Data on T3 EIA. Quantification of T3 plasma levels in zebrafish after oral administration of the hormone according to the fish gender. See material and method for details.
(XLSX)

S3 Text Data on gene expression levels after 7 days of treatment. Expression levels of melanophore-related genes after 7 days of T3 treatment according to fish gender as measured by qPCR. See material and method for details.
(XLSX)

S4 Text Data on gene expression levels after 15 days of treatment. Expression levels of melanophore-related genes after 15 days of T3 treatment according to fish gender as measured by qPCR. See material and method for details.
(XLSX)
