# Supplementary material for: Thyroid Hormones Regulate Zebrafish Melanogenesis in a Gender-Specific Manner
Source: PLoS One. 2016 Nov 10;11(11):e0166152. doi: 10.1371/journal.pone.0166152 (PMC5104317; doi:10.1371/journal.pone.0166152)
Supplement: S1 Table — Primer sequences used for quantification of pigmentation-related genes in zebrafish. Asip1 (agouti-signaling protein 1), Dct (dopachorme tautomerase or tyrosinase-related protein 2), FoxD3 (forkhead box D3), cKit (kit receptor tyrosine kinase type a or CD117), Mc1r (melanocortin receptor type 1), Mitfa (microphthalmia-associated transcription factor type a), Slc24a5 (solute carrier transporter 24a member 5), Sox10 (transcription factor Sox-10), Tyr (tyrosinase), Tyrp1a (tyrosinase-related protein 1 type a), Tyrp1b (tyrosinase-related protein 1 type b). See material and method for details. (DOCX) [file pone.0166152.s001.docx]

**S1 Table: Primers used for qPCR**

| **Name** | **Sequence** |
| --- | --- |
| Zf_qPCR_ASIP_Fw | 5' - CTGTGGGCGAGCTGCAAGAG - 3' |
| Zf_qPCR_ASIP_Rv | 5' - GCAGGGCTCCATAAACAGGAT - 3 |
| Zf_qPCR_Dct_Fw | 5' - ATGAAATGGCTCCAATCGGAC - 3' |
| Zf_qPCR_Dct_Rv | 5' - CAACACCAACACGATCAACAGC - 3' |
| Zf_qPCR_Foxd3_Fw | 5' -TTATCAAATCCGAGCCGTCCAG - 3' |
| Zf_qPCR_Foxd3_Rv | 5' - TGATATTTGACGGGACGCTGAG -3' |
| Zf_qPCR_c-Kita_Fw | 5' - TGCCCATGCAACAGAGAAAGAG -3' |
| Zf_qPCR_c-Kita_Rv | 5' - AAGTTCAACAGGTCGCCGAAAC - 3' |
| Zf_qPCR_c-Kitb_Fw | 5' - TCAAGTGGCTAAAGGCATGGAC - 3' |
| Zf_qPCR_c-Kitb_Rv | 5' - TTCACTGGCAGACGTGCATTTC - 3' |
| Zf_qPCR_MC1R_Fw | 5' - TCCCACAAACCCTTACTGCAAG - 3' |
| Zf_qPCR_MC1R_Rv | 5' - TACACTGCAAAGCACCACGAAC - 3' |
| Zf_qPCR_MITFa_Fw | 5' - TGCAGAAAGAGCAGCAGAAAGC - 3' |
| Zf_qPCR_MITFa_Rv | 5' - CAGGCTCTTGTTTGATTGCTCG - 3' |
| Zf_qPCR_slc24a5_Fw | 5' - TGATGGGAATGACTCTTCTGGC - 3' |
| Zf_qPCR_slc24a5_Rv | 5' - AAACACCGTCTGGATGAACCAC - 3' |
| Zf_qPCR_Sox10_Fw | 5' - GCACCACAATCGACACAAACTG - 3' |
| Zf_qPCR_Sox10_Rv | 5' - TTCAGGAAGGATGGATGCACTC - 3' |
| Zf_qPCR_TYR_Fw | 5' - TACTTCATGGTGCCCTTCATCC -3' |
| Zf_qPCR_TYR_Rv | 5' - CCGATGCGATTATTCCTGCTAC - 3' |
| Zf_qPCR_TYRP1a_2_Fw | 5' - CTGCCGTTCTGGGATTTCTC - 3' |
| Zf_qPCR_TYRP1a_2_Rv | 5' - GCTGGAGAAGATGGAGTTGGAG - 3' |
| Zf_qPCR_TYRP1b_Fw | 5' - CGACAACCTGGGATACACCT - 3' |
| Zf_qPCR_TYRP1b_Rv | 5' - AACCAGCACCACTGCAACT - 3' |

Primer sequences used for quantification of pigmentation-related genes in zebrafish. *Asip1* (agouti-signaling protein 1), *Dct* (dopachorme tautomerase or tyrosinase-related protein 2), *FoxD3* (forkhead box D3), *cKit* (kit receptor tyrosine kinase type a or CD117), *Mc1r* (melanocortin receptor type 1) *Mitfa* (microphthalmia-associated transcription factor type a), *Slc24a5* (solute carrier transporter 24a member 5), *Sox10* (transcription factor Sox-10), *Tyr* (tyrosinase), *Tyrp1a* (tyrosinase-related protein 1 type a), *Tyrp1b* (tyrosinase-related protein 1 type b).
