# Supplementary material for: Thyroid Hormones Regulate Zebrafish Melanogenesis in a Gender-Specific Manner
Source: PLoS One. 2016 Nov 10;11(11):e0166152. doi: 10.1371/journal.pone.0166152 (PMC5104317; doi:10.1371/journal.pone.0166152)
Supplement: S2 Table — Two-Way ANOVA table for comparisons on the number of melanophores in the Dorsal 2 (D2), Ventral 1 (V1), Ventral 2 (V2) or Dorsal 2 + Ventral 1+Ventral 2 (D2+V1+V2) stripes after T3 treatment according to fish gender. Differences (bold numbers) were considered significant when p<0.01 after Bonferroni's correction for multiple tests. See material and method for details. (DOCX) [file pone.0166152.s002.docx]

**S2 Table. Comparisons on the number of melanophores**

|  | D2 | V1 | V2 | D2+V1+V2 |
| --- | --- | --- | --- | --- |

|  | *SS* | *p* | *SS* | *p* | *SS* | *p* | *SS* | *p* |
| --- | --- | --- | --- | --- | --- | --- | --- | --- |
| **Sex** | 2553.7 | **<**0.039 | 3123.9 | **<0.001** | 1574.4 | **<0.001** | 21349.1 | **<0.001** |
| **T3** | 16556.5 | **<0.001** | 19357.3 | **<0.01** | 12978.8 | **<0.001** | 145791.9 | **<0.001** |
| **Interaction** | 85 | 0.70 | 552.7 | 0.144 | 917.6 | **<0.01** | 2267.5 | 0.1897 |

Two-Way ANOVA table for comparisons on the number of melanophores in the Dorsal 2 (D2), Ventral 1 (V1), Ventral 2 (V2) or Dorsal 2 + Ventral 1+Ventral 2 (D2+V1+V2) stripes after T3 treatment according to fish gender. Differences (bold numbers) were considered significant when p<0.01 after Bonferroni's correction for multiple tests.
