# Supplementary material for: Thyroid Hormones Regulate Zebrafish Melanogenesis in a Gender-Specific Manner
Source: PLoS One. 2016 Nov 10;11(11):e0166152. doi: 10.1371/journal.pone.0166152 (PMC5104317; doi:10.1371/journal.pone.0166152)
Supplement: S3 Table — Two-Way ANOVA table for comparisons on the expression of melanophore-related genes after 7 and 15 days of T3 treatment according to fish gender. Differences (bold numbers) were considered significant when p<0.004 after Bonferroni's correction for multiple tests. See material and method for details. (DOCX) [file pone.0166152.s003.docx]

**S3 Table. Comparisons on the expression of melanophore-related genes**

|  | **ASIP** | **Dct/Tyrp2** | **Foxd3** | **Kita** | **Kitb** | **MC1R** | **Mitfa** | **Slc24a5** | **Sox10** | **Tyr** | **Tyrp1a** | **Tyrp1b** |
| --- | --- | --- | --- | --- | --- | --- | --- | --- | --- | --- | --- | --- |

**7 Days**

|  | *SS* | *p* | *SS* | *p* | *SS* | *p* | *SS* | *p* | *SS* | *p* | *SS* | *p* | *SS* | *p* | *SS* | *p* | *SS* | *p* | *SS* | *p* | *SS* | *p* | *SS* | *p* |
| --- | --- | --- | --- | --- | --- | --- | --- | --- | --- | --- | --- | --- | --- | --- | --- | --- | --- | --- | --- | --- | --- | --- | --- | --- |
| **Sex** | 3.47 | 0.04 | 27.2 | **<0.004** | 0.18 | 0.94 | 0 | 1 | 1.67 | 0.20 | 1.79 | 0.13 | 3.93 | 0.02 | 30.83 | **<0.004** | 4.38 | **<0.004** | 1.11 | 0.18 | 0.04 | 0.84 | 0.01 | 0.92 |
| **T3** | 0.21 | 0.6 | 5.72 | 0.017 | 1.29 | 0.45 | 0.49 | 0.31 | 3.47 | 0.07 | 4.61 | 0.02 | 0 | 1 | 2.09 | 0.29 | 0.55 | 0.33 | 11.26 | **<0.004** | 0.22 | 0.65 | 9.86 | **<0.004** |
| **Interaction** | 0.04 | 0.82 | 0.3 | 0.565 | 0.09 | 0.66 | 2.44 | 0.02 | 0 | 1 | 0.55 | 0.40 | 1.22 | 0.17 | 1.8 | 0.33 | 0.87 | 0.22 | 6.41 | **<0.004** | 0.21 | 0.66 | 0.41 | 0.46 |

**15 Days**

| **Sex** | 5.5 | **<0.004** | 101.3 | **<0.004** | 0.02 | 0.843 | 1.66 | **<0.004** | 0.32 | 0.50 | 6.58 | **<0.004** | 10.2 | **<0.004** | 19.2 | **<0.004** | 0.43 | 0.33 | 13.47 | **<0.004** | 0.19 | 0.325 | 2.05 | 0.12 |
| --- | --- | --- | --- | --- | --- | --- | --- | --- | --- | --- | --- | --- | --- | --- | --- | --- | --- | --- | --- | --- | --- | --- | --- | --- |
| **T3** | 0.06 | 0.65 | 2.87 | 0.08 | 9.48 | **<0.004** | 0.13 | 0.38 | 0.92 | 0.26 | 0.16 | 0.47 | 1.51 | 0.23 | 2.08 | 0.18 | 1.78 | 0.05 | 2.54 | 0.07 | 1.14 | 0.02 | 51.04 | **<0.004** |
| **Interaction** | 0.9 | 0.08 | 20.2 | **<0.004** | 0.57 | 0.307 | 0.42 | 0.12 | 3.98 | 0.02 | 1.62 | 0.028 | 5.84 | 0.02 | 1.98 | 0.19 | 0.28 | 0.42 | 10.24 | **<0.004** | 0.13 | 0.41 | 4.38 | 0.03 |

Two-Way ANOVA table for comparisons on the expression of melanophore-related genes after 7 and 15 days of T3 treatment according to fish gender. Differences (bold numbers) were considered significant when p<0.004 after Bonferroni's correction for multiple tests
